# TREM2 regulates innate immunity in Alzheimer’s disease

**DOI:** 10.1186/s12974-018-1148-y

**Published:** 2018-04-14

**Authors:** Jiang-Tao Li, Ying Zhang

**Affiliations:** College of Life Sciences and Bioengineering, Beijing Jiaotong University, No. 3 Shangyuan Residence, Haidian District, Beijing, 100044 China

**Keywords:** Alzheimer’s disease (AD), Triggering receptor expressed on myeloid cells 2 (TREM2), Innate immunity, Inflammation

## Abstract

Recent research has shown that the triggering receptor expressed on myeloid cells 2 (TREM2) in microglia is closely related to the pathogenesis of Alzheimer’s disease (AD). The mechanism of this relationship, however, remains unclear. TREM2 is part of the TREM family of receptors, which are expressed primarily in myeloid cells, including monocytes, dendritic cells, and microglia. The TREM family members are cell surface glycoproteins with an immunoglobulin-like extracellular domain, a transmembrane region and a short cytoplasmic tail region. The present article reviews the following: (1) the structure, function, and variant site analysis of the *Trem2* gene; (2) the metabolism of TREM2 in peripheral blood and cerebrospinal fluid; and (3) the possible underlying mechanism by which TREM2 regulates innate immunity and participates in AD.

## Background

Alzheimer’s disease (AD) is the most common age-related neurodegenerative disease. The early symptoms of AD are short-term memory loss and disorientation, followed by progressive memory loss and irreversible cognitive decline. As AD progresses, severe clinical neuropsychiatric symptoms appear, and the patients can no longer take care of themselves. On average, a person with Alzheimer’s lives 4 to 8 years after diagnosis, but patients can live as long as 20 years, depending on other factors. AD is characterized by an abnormal aggregation of β-amyloid (Aβ) peptides and neuronal neurofibrillary tangles (NFTs) derived from hyperphosphorylated tau (p-tau).

Currently, approximately 47 million people live with dementia worldwide, and that number will increase to more than 131 million by 2050 [1]. The global costs of AD will increase to $1 trillion by 2018. Therefore, AD has become an urgent health problem around the world [1].

Innate immunity is a type of non-targeted defense mechanism [2]. When a living organism makes contact with the external environment, such as with viruses, germs, or other pathogenic microorganisms, innate immunity can protect and keep our bodies healthy. Innate immunity has been gradually established during the long-term process of evolution. Over the past few years, genetic research has found some new pathogenic factors associated with AD [3]. In the analysis of these factors, the innate immune system has attracted a great deal of attention, especially regarding the function of microglia [4].

Microglia are macrophages in the brain and spinal cord and act as the first line of immune defense in the central nervous system (CNS). Microglia participate in the identification of pathogens and activate the innate immune response, which is of major importance in the brain [5]. Based on previous reports, we know that mouse microglial cells exhibit a chemotactic response to β-amyloid 1-42 (Aβ42). Furthermore, mouse homolog formyl peptide receptor 2 (mFPR2) enhances Aβ42 internalization when the microglia are stimulated by Toll-like receptors (TLRs) [6]. The activation of TLRs promotes the ability of microglia to digest and process Aβ42. In the pathologic process of AD, the clearance of microglial cells may be dynamic [6, 7]. Triggering receptor expressed on myeloid cells 2 (TREM2) is expressed in microglia. Studies [8, 9] have shown that certain TREM2 variants have an important effect on AD, and that effect is similar to that of apolipoprotein E (ApoE). They are all the risk factors of AD. TREM2 and ApoE ε4 may interact synergistically in the preclinical stage of AD [10].

## Structure and function of the *Trem2* gene

**Fig. 1:**
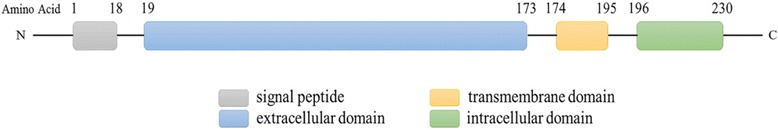
Diagrams of the structure of TREM2. The protein structure has 230 amino acids (aa). TREM2 includes a signal peptide (aa1-18, gray), a transmembrane region (aa174-195, orange), an extracellular domain (aa19-173, blue), and an intracellular domain (aa196-230, green)

The *Trem2* gene is located on human chromosome 6, from 41,126,246 bp to 41,130,922 bp, with a total length of 4676 bp. TREM2 consists of five exons that can encode a 230 amino acid protein (Fig. 1) [11]. TREM2 belongs to the TREM family of receptors, which are expressed in a variety of myeloid cells. TREM2 is mainly expressed in monocytes, macrophages, dendritic cells, and microglia. Members of the TREM family are cell surface glycoproteins with immunoglobulin-like extracellular domains, transmembrane regions, and short cytoplasmic tails. In the brain, TREM2 is involved in regulating the inflammatory responses of microglia and phagocytosis of cellular debris.

A decade ago, TREM2 has been founded as a phagocytic receptor for bacteria [12]. In neural cells, TREM2 signaling is completely dependent on the adapter protein, DNAX-activation protein 12 (DAP12, also known as TYROBP), because the major isoform of TREM2 has a short cytoplasmic tail. Since TREM2 lacks an additional cytoplasmic domain, TREM2 must signal via DAP12, which contains an immunoreceptor tyrosine-based activation motif (ITAM) [13]. This cooperation is absolutely necessary for effective phagocytosis.

While studies have suggested that TREM2 can regulate the number of myeloid cells, the impact of this occurrence in AD remains unknown. TREM2 knockdown in primary microglia was found to reduce cell number [14], while crosslinking TREM2 promoted an increase in osteoclast number in cell cultures [15]. It has been confirmed that TREM2 can increase the number of myeloid cells in the context of inflammation or disease. Recent studies have revealed that myeloid cell accumulation around amyloid plaques was reduced in TREM2 hemizygous [16, 17] and DAP12-deficient [17] AD mouse models.

Enhanced phagocytosis is an important function of TREM2. TREM2 is expressed in myeloid cells in the CNS, which have high phagocytic activity [18]. Both in vitro and in vivo studies have shown that a loss of TREM2 function results in reduced phagocytosis [19] and β-amyloid 1-42 (Aβ42) uptake [20]. In contrast, TREM2 overexpression by lentivirus vector system could enhance the clearance of apoptotic neurons [21].

### TREM2 variants in Alzheimer’s disease

**Fig. 2:**
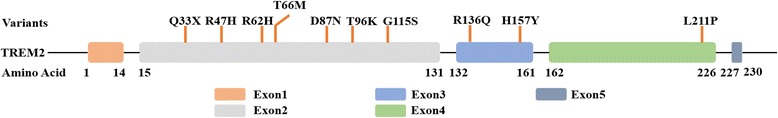
Diverse *TREM2* variants are associated with AD. There are five exons: exon 1(aa1-14, orange), exon 2 (aa15-131, gray), exon 3 (aa132-161, blue), exon 4 (aa 162-226, green), and exon 5 (aa227-230, turquoise blue). Genetic variants of the *TREM2* gene result in diverse changes in the protein structure (shown above)

Studies have shown that a few rare variants of TREM2 are considered to be associated with susceptibility to AD [22, 23]. Research on TREM2 missense mutations in European populations revealed that variants, such as L211P [24], H157Y [25], R136Q [26], T96K [26], D87N [27], T66M [26], R62H [28], R47H [27], and Q33X [29] (Fig. 2), have been found to be associated with AD. However, studies of non-European populations have shown different results. In our research on the Chinese population, the R47H missense variant was very rare, and another missense variant, G115S, was found to be related to AD [30].

Many studies reported that the R47H variant of TREM2 is associated with the risk of AD [9, 27, 31–33]. Lill and her colleagues reported that the rs75932628 variant of TREM2 significantly increased the level of CSF-total-tau but not Aβ42 in a European population and suggested that the role of TREM2 in AD may involve tau dysfunction [34]. However, as shown in previous studies, the rs75932628 variant of TREM2 was not detected in either Chinese or Korean populations [30, 35–37]. These results suggest that TREM2 is differentially associated with the incidence of AD in varying ethnicities, which may be related to the genetic backgrounds of different races.

The R47H variant of TREM2 increases terminal glycosylation of complex oligosaccharides in the Golgi apparatus and reduces TREM2’s solubility. This may affect the binding of DAP12 to TREM2, which would, in turn, affect the function of the receptor [38]. Meanwhile, the R47H variant has been presumed to destroy the stability of the TREM2 protein [39]. On the basis of crystalline structural analysis, another explanation suggested that AD risk variant R47H might impact binding to a cell-surface ligand (TREM2-L) and slightly impact the stability and structure of TREM2 [40]. However, a contrary result showed that transfected R47H-TREM2 constructs have an increased half-life relative to wild-type TREM2 and can resist proteasome degradation in the endoplasmic reticulum (ER) [38]. These statements are possible, which depend on the specific activity of life. The tyrosine-38 and threonine-66 residues of TREM2 are essential for the glycosylation of the protein. The Y38C and T66M variants of TREM2 may cause some significant differences in the glycosylation patterns and damage during transport to the plasma membrane. In the previous studies, it was found that the TREM2 R47H variant had a slight difference in N-glycosylation of the complex oligosaccharide compared to the Y38C and T66M variants, which are associated with Nasu-Hakola disease (NHD) [38]. This difference causes NHD to be an early-onset disease and AD to be a late-onset disease.

### TREM2 expression and regulation in Alzheimer’s disease

Data have shown that TREM2 plays a vital role in the cognitive function of the brain. An important function of TREM2 is its regulation of phagocytosis in microglia. Microglial removal of damaged cells, organic matrix molecules, and biomacromolecules must be assisted by the TREM2-DAP12 receptor complex. As a glial cell immunoreceptor, TREM2 has been found to modulate microglia-mediated inflammatory responses [41]. A decade ago, Gordon described the mechanism of two opposite types of macrophage activation [42], but now, the type M1 and M2 are widely used to define classically (proinflammatory) and alternatively activated (anti-inflammatory) microglia, which is controversial [43]. Outside the CNS, the mononuclear phagocyte system has been divided into M1 phenotype and M2 phenotype. Study shows that in the CNS, because that microglial activation is heterogeneous, the microglia can also be categorized into two opposite types: M1 phenotype and M2 phenotype [44]. Therefore, we conjecture that in the brain, microglia have two opposite roles, proinflammatory (M1, cytotoxic) and anti-inflammatory (M2, neuroprotective). TREM2 inhibits neurotransmitters by blocking M2 microglia. This may reveal the potential mechanism by which TREM2 inhibits microglial inflammatory responses [45]. The microglial cells participate in the removal of Aβ aggregates through phagocytosis. Previous study found that TREM2 overexpression by intracerebral lentiviral particle injection significantly reduced soluble and insoluble Aβ42 aggregates in the brain. In middle-aged APPswe/PS1ΔE9 mice (7–8 months old), the ability of microglia to remove amyloid plaques increased after TREM2 overexpression, and the density of amyloid plaques in the brain decreased [20]. However, in a mouse model of TREM2 defects, the concentration of amyloid plaques in the brain did not change [46]. Interestingly, after the expression of TREM2 in 18-month-old APPswe/PS1ΔE9 mice, the concentration of amyloid plaques was not attenuated, and no alterations in the levels of Aβ42 were observed in the brain [46]. Research utilizing mouse models has shown that the overexpression of TREM2 plays a protective role in both early- and mid-term AD, whereas this protective effect is lost in late-term AD [47]. We speculate that the reduced number of microglia in the brains of older mice may lead to a decline in phagocytosis.

The high level of phosphorylation and abnormal aggregation of tau protein are pathophysiological factors associated with neuronal and synaptic damage. The loss of neurons and synapses in the hippocampus is associated with a decrease in spatial cognitive function. The 7-month-old P301S mouse model has been shown to exhibit significant neuronal and synaptic damage to this region. Overexpression of TREM2 is effective in inhibiting these lesions; water maze experiments have demonstrated that TREM2 overexpression can restore spatial cognitive impairment in mice [47]. In addition, the overexpression of TREM2 by intracerebral lentiviral particles injection has been found to significantly improve hyperphosphorylation of tau proteins and reduce the activity of cyclin-dependent kinase 5 (CDK5) and glycogen synthase kinase-3β (GSK3β) [45]. Thus, TREM2 overexpression significantly reduces neuronal loss and may play a role in the phosphorylation of tau protein, thereby reducing the incidence of AD.

A recent study showed that TREM2 releases its extracellular domain after protease cleavage, leaving only the carboxy-terminal fragment (CTF) attached to the membrane [48]. Soluble TREM2 (sTREM2) may be produced by proteolytic cleavage and alternative splicing. If insertions [49] or frameshifts [50] occur in exon 4, it can terminate the transmembrane domain, which is speculated to yield a soluble product. In addition to the membrane-bound form, sTREM2 has been detected in the supernatants of human [51] and mouse [51] cell cultures and in the peripheral blood and cerebrospinal fluid (CSF) [52]. The sTREM2 in human peripheral blood and CSF can be used as a more accurate tool for understanding the biological effects of TREM2 in the pathogenesis of AD. Hu et al. analyzed the expression of TREM2 mRNA and protein in the peripheral blood in a population of Northern Han Chinese [53]. The results showed that on the level of mRNA and protein, TREM2 expression were higher on monocytes, granulocytes, and in plasma in AD group compared with that of control groups. Mori et al. performed a similar analysis of TREM2 expression in the peripheral blood in a small population of Japanese individuals (26 patients with AD, 8 males and 18 females) [54]. However, another study mentioned that the absolute level of TREM2 expression in human peripheral blood monocytes is quite low and unlikely to be useful for drawing mechanistic conclusions about TREM2 [55]. The upregulation of TREM2 in the peripheral blood indicates that the gene is abnormally active in the development of AD pathology. More experiments are needed to confirm whether TREM2 was differently expressed in the peripheral blood in some populations. The level of sTREM2 in the CSF also exhibits changes. Although Kleinberge et al. showed that sTREM2 levels were reduced in the CSF of AD patients [56], other studies have shown that sTREM2 levels in the CSF increased with age and were positively correlated with the levels of Aβ42 and tau protein [26, 57–59].

### TREM2 modulates inflammatory responses

While most people think that TREM2 exerts an anti-inflammatory effect, it seems that the connection between TREM2 and other inflammatory responses is not so simple. According to the cell type and context, the strength [60] and duration [61] of the stimuli is different. Therefore, TREM2 seems to play different roles in inflammatory responses.

Some in vitro and in vivo studies have shown that TREM2 plays an anti-inflammatory role in certain contexts. In cell lines, TREM2 deficiency increases the levels of proinflammatory mediators, such as tumor necrosis factor-α (TNFα), interleukin-1β (IL1β), and interleukin-6 (IL6) [62]. TREM2 knockdown in the senescence-accelerated mouse P8 (SAMP8) mouse model also increased the production of inflammatory cytokines [63]. Furthermore, overexpressing TREM2 in AD mouse models [20, 45] reduced the levels of proinflammatory transcripts. From these studies, we can speculate that TREM2 can inhibit inflammatory responses in some contexts.

However, many studies have supported that TREM2 can amplify or promote inflammatory responses. TREM2-deficient microglia have reduced activation and a more ramified morphology in cell cultures [19]. In AD mouse models, TREM2-deficient microglia exhibit decreased cell size and surface area, as well as increased process length, resulting in reduced activation [16]. sTREM2 activate the Akt–GSK3β–β-catenin pathway, which can suppress apoptosis in microglia [64]. In this study [65], TREM2 promotes microglial survival by activating the Wnt/β-catenin signaling pathway. The upregulation of the Wnt/β-catenin pathway suppresses GSK3β, restores β-catenin signaling, and promotes TREM2-deficient microglial survival in vitro and in vivo. NF-κB signaling is associated with proinflammatory cytokines; the inhibition of NF-κB signaling markedly downregulated the production of three proinflammatory cytokines (IL-1β, IL-6, and TNF) [64]. Taken together, these findings clearly supported that TREM2 can regulate inflammatory responses.

### TREM2-mediated neuroprotection in microglia

Aβ can destroy synaptic transmission, induce oxidative stress, and trigger cell death in vitro [66]. Meanwhile, microglia have been shown to devour Aβ in the brain [67]. Therefore, microglial phagocytosis of Aβ may serve a neuroprotective function. However, the absence of TREM2 significantly impairs the ability of microglia to engulf amyloid plaques. Some studies have reported that a TREM2-deficient AD mouse model results in a decrease in the number of microglia around amyloid plaques because metabolic fitness is reduced [68].

Condello et al. proposed a new hypothesis [69]; they postulated that the tight envelope of microglia around the amyloid surface constitutes a neuroprotective barrier that limits fibril outgrowth and plaque-associated toxicity. In AD mouse models, a lack of TREM2 or DAP12 results in more dispersed amyloid plaques and increased synapses, causing a morphology that resembles a sea urchin [17]. The greater the number of synapses that protrude outside is, the larger the contacted surface with nerve structures and the greater the potential harm to the nervous system. Thus, in human brains, the protective role of microglia may primarily act as a barrier that isolates amyloid plaques from peripheral nerve tissues.

## Conclusion

**Fig. 3:**
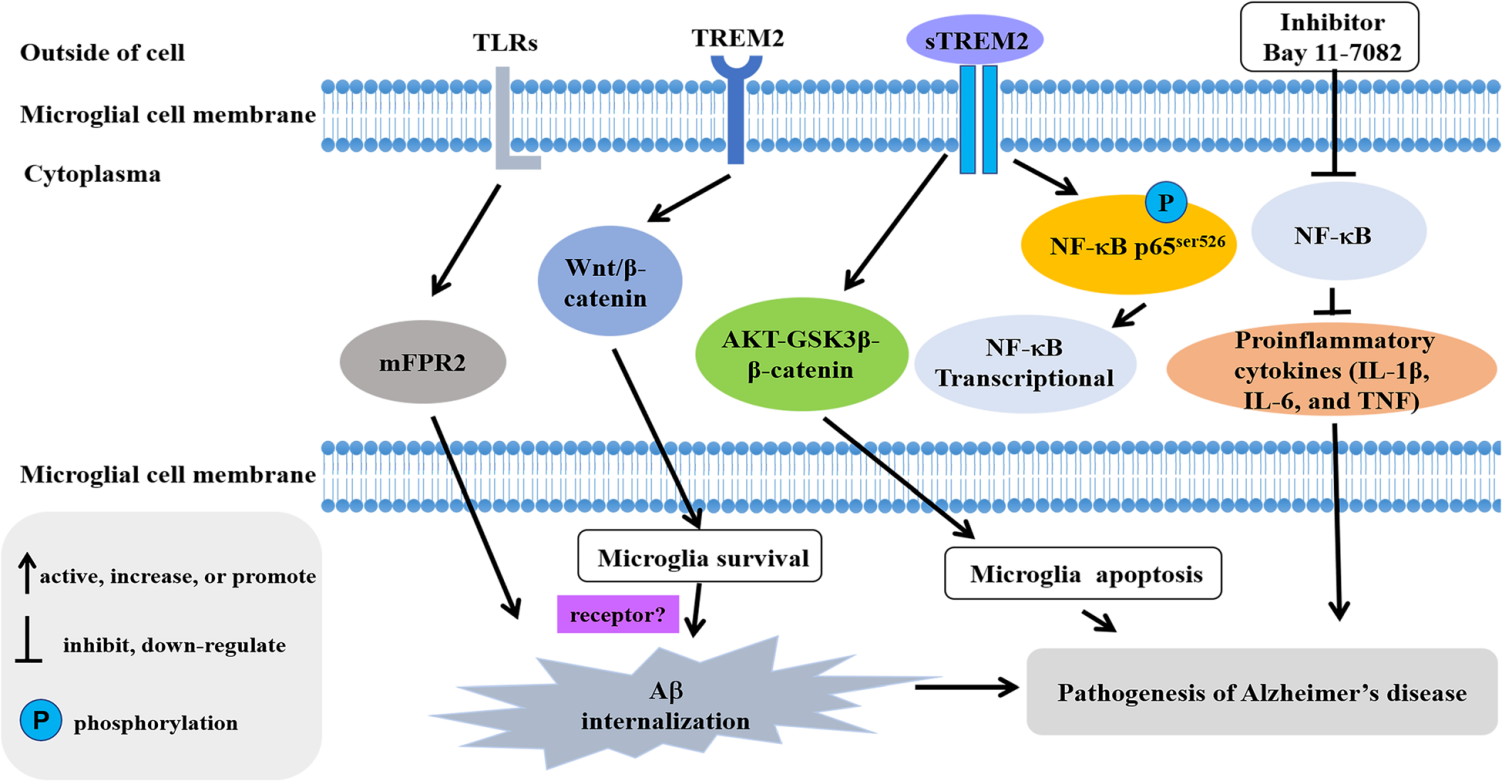
Schematic illustration of TREM2 mediated innate immune regulation in Alzheimer’s disease. The pathways include (1) Wnt/β-catenin signal pathway, (2) AKT-GSK3β-β-catenin, and (3) NF-κB signal pathway

The relationships between TREM2 and *TREM2* gene expression, function, mutation site analysis, and metabolism in peripheral blood and cerebrospinal fluid were reviewed in this paper. It is important to note that in the TREM family, the *Trem2* gene plays an important role in the pathogenesis of AD. TREM2 can maintain the ability of microglia to recover neurons and engulf damaged neurons. However, some variants of this gene not only lead to changes in TREM2 expression levels but also impact the ability of TREM2 to bind to its ligand in microglia [55, 70]. Thus, these gene variants can influence the natural immune system. TREM2 mediates the neuroprotection in microglial cells by regulating the inflammatory responses and microglia survival (Fig. 3).

These results indicate that TREM2 may be a potential biomarker for AD diagnosis and treatment. In addition, TREM2 missense mutants have been found in many neurological immune deficiencies, indicating that TREM2 variants impact the immune function of the nervous system. Further research is needed to elucidate the biological role of TREM2 in the natural immune regulation of Alzheimer’s disease. Therefore, it is important to understand when, where, and how TREM2 plays a role in AD. This information could possibly provide new insights into immune function and immunotherapy, such that we could regulate this disease throughout its progression.

## Abbreviations

AD: Alzheimer’s disease
ApoE: Apolipoprotein E
Aβ: β-amyloid
Aβ42: β-Amyloid 1-42
CDK5: Cyclin-dependent kinase 5
CNS: Central nervous system
CSF: Cerebrospinal fluid
DAP12: DNAX-activation protein 12
ER: Endoplasmic reticulum
GSK3β: Glycogen synthase kinase-3β
IL1β: Interleukin-1β
IL6: Interleukin-6
ITAM: Immunoreceptor tyrosine-based activation motif
mFPR2: Mouse homolog formyl peptide receptor 2
NFTs: Neurofibrillary tangles
NHD: Nasu-Hakola disease
p-tau: Hyperphosphorylated tau
SAMP8: Senescence-accelerated mouse P8
sTREM2: Soluble TREM2
TLR: Toll-like receptor
TNFα: Tumor necrosis factor-α
TREM: Triggering receptor expressed on myeloid cells
TREM2: Triggering receptor expressed on myeloid cells 2
